# Induced genetic diversity through mutagenesis in wheat gene pool and significant use of SCoT markers to underpin key agronomic traits

**DOI:** 10.1186/s12870-024-05345-5

**Published:** 2024-07-15

**Authors:** Ahmed Ali Abdelhameed, Mohammed Ali, Doaa Bahaa Eldin Darwish, Manal Abdullah AlShaqhaa, Dalia Abdel-Fattah H. Selim, Aziza Nagah, Muhammad Zayed

**Affiliations:** 1 Agricultural Botany Department (Genetics), Faculty of Agriculture, Al-Azhar University, Assuit Branch, Assuit, 71524 Egypt; 2 Maryout Research Station, Genetic Resources Department, Desert Research Center, 1 Mathaf El-Matarya St., El-Matareya, Cairo, 11753 Egypt; 3 Faculty of Science, University of Tabuk, Tabuk, 71491 Saudi Arabia; 4 Department of Biology, College of Science, King Khalid University, Abha, 61413 Saudi Arabia; 5 Department of Agricultural Botany, Faculty of Agricultural, Menoufia University, Shebin El-Kom, 32511 Egypt; 6 Botany and Microbiology Department, Faculty of Science, Benha University, Benha, 13518 Egypt; 7 Department of Botany and Microbiology, Faculty of Science, Menoufia University, Shebin El-Kom, 32511 Egypt

**Keywords:** Wheat (*Triticum aestivum* L.), Chemical mutagenesis, Genetic diversity, Gene pool, Sodium azide, Hydrazine hydrate, SCoT marker, Agronomic traits, Polymorphism

## Abstract

**Background:**

This research explores the efficacy of mutagenesis, specifically using sodium azide (SA) and hydrazine hydrate (HZ) treatments, to introduce genetic diversity and enhance traits in three wheat (*Triticum aestivum* L.) genotypes. The experiment entails subjecting the seeds to different doses of SA and HZ and cultivating them in the field for two consecutive generations: M1 (first generation) and M2 (second generation). We then employed selective breeding techniques with Start Codon Targeted (SCoT) markers to select traits within the wheat gene pool. Also, the correlation between SCoT markers and specific agronomic traits provides insights into the genetic mechanisms underlying mutagenesis-induced changes in wheat.

**Results:**

In the study, eleven genotypes were derived from parent varieties Sids1, Sids12, and Giza 168, and eight mutant genotypes were selected from the M1 generation and further cultivated to establish the M2 generation. The results revealed that various morphological and agronomical characteristics, such as plant height, spikes per plant, spike length, spikelet per spike, grains per spikelet, and 100-grain weight, showed increases in different genotypes from M1 to M2. SCoT markers were employed to assess genetic diversity among the eleven genotypes. The bioinformatics analysis identified a correlation between SCoT markers and the transcription factors ABSCISIC ACID INSENSITIVE3 (ABI3) and VIVIPAROUS1 (VP1), crucial for plant development, growth, and stress adaptation. A comprehensive examination of genetic distance and the function identification of gene-associated SCoT markers may provide valuable insights into the mechanisms by which SA and HZ act as mutagens, enhancing wheat agronomic qualities.

**Conclusions:**

This study demonstrates the effective use of SA and HZ treatments to induce gene diversity through mutagenesis in the wheat gene pool, resulting in the enhancement of agronomic traits, as revealed by SCoT markers. The significant improvements in morphological and agronomical characteristics highlight the potential of mutagenesis techniques for crop improvement. These findings offer valuable information for breeders to develop effective breeding programs to enhance wheat quality and resilience through increased genetic diversity.

**Supplementary Information:**

The online version contains supplementary material available at 10.1186/s12870-024-05345-5.

## Background

Genetic diversity eases adaptive evolutionary changes that build up the genetic improvement of any crop plant, and mutation is the primary driver of all genetic diversity. Mutations occur naturally but at a meager rate. For example, the in vivo mutation rate is less than one mistake for every billion base pairs copied during replication [1, 2]. The buildup of unrepaired DNA damage can alter the genotype and phenotype of somatic and germline cells. Therefore, induced mutations may successfully invigorate genetic diversity in crops [3, 4].

Many methods, such as radiation, chemical mutagens, hybridization, and transposon insertion, can induce mutation. Mutagenic chemicals (mutagens), such as alkylating or intercalating agents, can change DNA bases randomly. Hydrazine hydrate (HZ), Sodium azide (SA), ethyl methane sulphonate (EMS), ethyleneimines, alkyl methane sulphonates, sulfur mustards, methyl methane sulfonate, epoxides, and alkyl nitrosoureas are examples of chemicals that proved to induce mutations [5–10]. For example, mutagens produced highly diverged lines of Salvia (*Salvia officinalis*) [11], Chickpea (*Cicer arietinum* L.) [12], rice (*Oryza sativa*) [13–15], Arabidopsis (*Arabidopsis thaliana*) [16], Dianthus (*Dianthus caryophyllus*) [17], sweet corn (*Zea mays*) [18], and barley (*Hordeum vulgare* L.) [19].

Mutation breeding can rapidly introduce new mutations that may result in unique traits not present in the original germplasm [20]. However, traditional breeding methods depend on introducing genetic diversity via recombination and rearranging the available alleles, a process limited by the boundaries of the current gene pool. This limitation arises from the dependency on crossing plants with favorable traits, followed by selecting desirable individuals. Such selection pressure may result in losing certain qualities such as flavor, nutritional value, and resilience to biotic and abiotic stresses [21]. While mutant breeding may be performed quickly, the results need careful inspection since it often produces several off-target consequences. Conversely, conventional breeding is a gradual process noted for its predictability due to its focused trait selection approach [22, 23].

Chemical mutagens such as SA and HZ are quite effective in producing random point mutations across the genome [11, 24–27]. The effectiveness of these mutagens was estimated by calculating the number of mutations due to exposure to a unit dose of the mutagen. However, the ratio of the frequency of mutations caused to the biological damage is the measure of their mutagenic efficiencies [24].

Wheat is the leading universal source of calories. Therefore, to provide food security challenges, the genetic improvements of wheat to enhance yield and quality and improve its tolerance against biotic or abiotic stressors were targets of many governments [28, 29]. This study aims to harness SA and HZ as mutagens that induce random global mutations across the wheat *Triticum aestivum* genome to enhance its diversity and improve various wheat agronomic traits. The genetic variation can be assessed by various molecular markers such as Restriction Fragment Length Polymorphism (RFLP), Random Amplified Polymorphic DNA (RAPD), Start Codon Targeted (SCoT), Simple Sequence Repeat (SSR), Single Nucleotide Polymorphism (SNP), Inter Simple Sequence Repeat (ISSR), and Amplified Fragment Length Polymorphism (AFLP) [30–32]. Start Codon Targeted (SCoT) markers were chosen to assess the genetic diversity after mutation-inducing mutagens. SCoT markers are highly informative and provide an efficient tool to elucidate genetic differentiation among mutated wheat varieties. This study concludes the impact of mutagenesis on the genetic diversity of the wheat gene pool to enhance agronomic traits through harnessing SCoT markers to identify and characterize improved phenotypic expressions of some important agronomic traits in wheat. The correlation between SCoT markers and specific agronomic traits provides insights into the genetic mechanisms underlying mutagenesis-induced changes in wheat. Furthermore, the proposed functions and the putative tissue expression patterns of the candidate genes associated with the list of SCoT primers in this study were elucidated.

## Results

### Variation in morphological and agronomical attributes

**Fig. 1:**
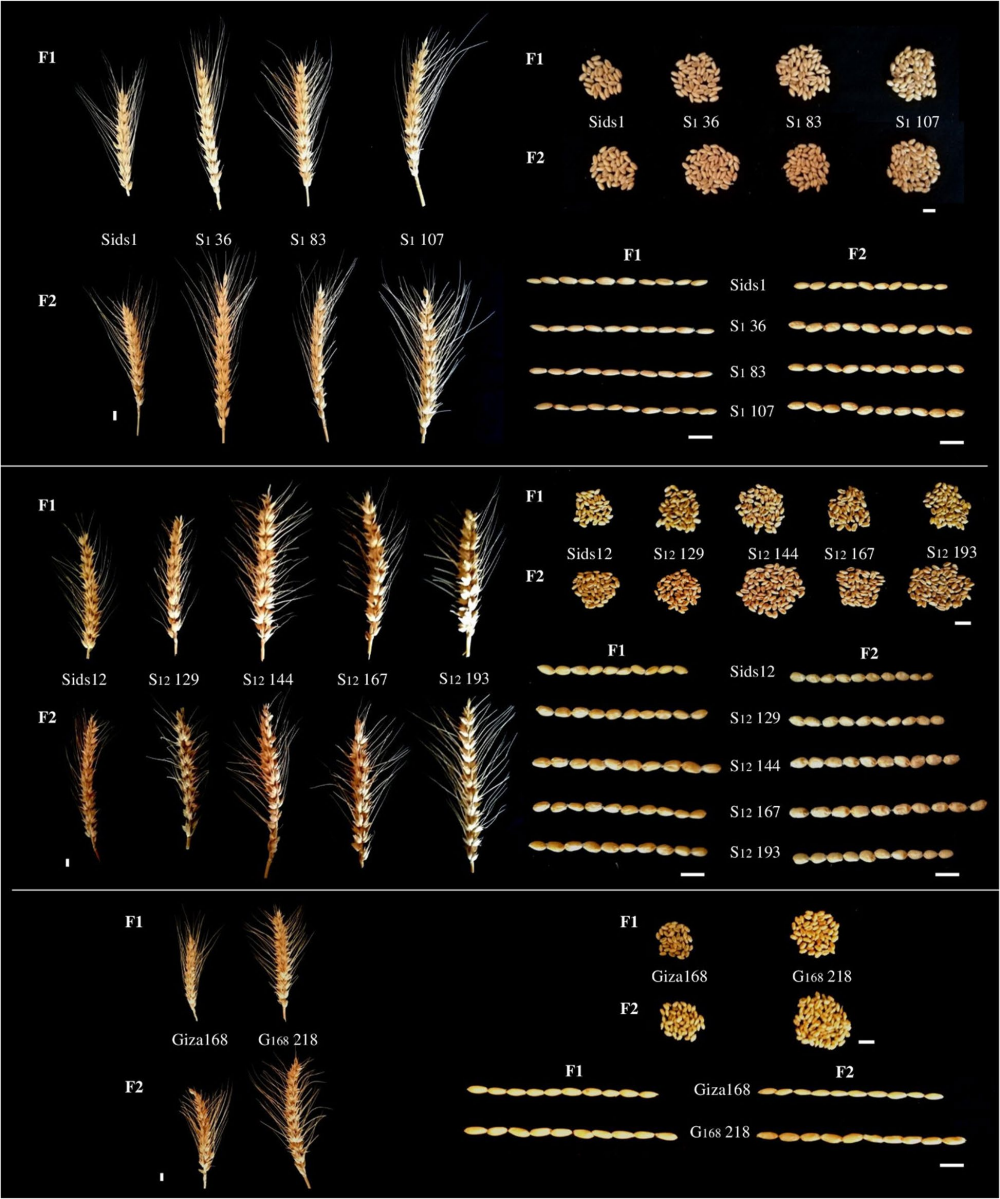
Morphological and agronomical attributes in the three varieties of wheat (Sids1, Sids12, and Giza168) and their mutants in the first and second generations as induced by SA and HZ treatments

The outcomes of the current investigation demonstrated broad selections expressed at six morphological and agronomical attributes in the three varieties of wheat (Sids1, Sids12, and Giza168) and their mutants (S 36, S 83, S 107, S 129, S 144, S 167, S 193, and G 218) at M1 and M2 generations as induced by SA and HZ treatments (Fig. 1 and Table 1). Sids1 and some of their mutants showed the highest mean values in the examined morphological and agronomical traits in the M2 generation compared with the M1 generation. For example, the highest mean plant height value (119.5 cm) was obtained in the S_1_83 genotype. Likewise, the most significant average value of the number (no.) of spikes/plant (22.4) was obtained in the S_1_36 genotype. Moreover, the highest mean spike length (18.8 cm) was achieved for the mutated genotype S_1_83. The maximum number of spikelets/spike (25.8) was found in both mutated genotypes S_1_83 and S_1_107. Furthermore, the highest no. of grains /spikelet (5.2) was recorded in both mutated genotypes S_1_36 and S_1_107. At the same time, the manalyze the genetic diversity of eleven wheat s compared to the control (3.8) (Fig. 1 and Table 1).

**Table 1:** Mean performance of the eleven genotypes at M1 and M2 generations for all the studied morphological and agronomical traits in the 2019 /2020 and 2020/2021 seasons. Sids1 and their three mutant genotypes (S 36, S 83 and S 107), Sids12 and their four mutant genotypes (S 129, S 144, S 167 and S 193) and Giza 168 and its mutant genotype (G 218)

Genotype	Treatment		Plant height	No. spikes/plant	Spike length	No. spickelet/spike	No. grains/spikelet	100 grain weight
**Sids1**								
** Sids1**	**Control**	M1	103.03 ± 0.50^c^	7.33 ± 1.15^c^	13.06 ± 0.61^c^	20.0 ± 4.0^b^	3.33 ± 1.15^b^	4.13 ± 0.41^c^
		M2	100.23 ± 1.21^d^	6.6 ± 2.14^d^	13.2 ± 0.69^d^	18.8 ± 2.06^c^	3.1 ± 0.63^b^	3.8 ± 0.53^c^
** S**_**1**_** 36**	**0.025 SA**	M1	109.03 ± 0.50^b^	21.0 ± 2.0^a^	17.53 ± 0.70^ab^	24.0 ± 4.0^a^	5.0 ± 2.0^a^	5.39 ± 0.14^b^
		M2	108.8 ± 0.82^b^	22.4 ± 2.34^a^	18.4 ± 0.41^b^	24.4 ± 1.68^b^	5.2 ± 0.84^a^	5.43 ± 0.53^b^
** S**_**1**_** 83**	**0.0125 HZ**	M1	117.9 ± 1.9^a^	18.3 ± 1.15^b^	17.03 ± 0.70^b^	26.0 ± 4.0^a^	6.0 ± 2.0^a^	5.8 ± 0.50^ab^
		M2	119.5 ± 1.08^a^	20.8 ± 1.57^b^	18.8 ± 0.65^a^	25.8 ± 3.50^a^	5.1 ± 1.13^a^	5.42 ± 0.48^b^
** S**_**1**_** 107**	**0.025 HZ**	M1	108.4 ± 103^b^	20.0 ± 2.0^a^	18.03 ± 1.10^a^	22.0 ± 4.0^ab^	5.33 ± 1.15^a^	6.3 ± 0.8^a^
		M2	107.7 ± 0.67^c^	19.0 ± 1.88^c^	18.1 ± 0.54^c^	25.8 ± 2.27^a^	5.2 ± 1.57^a^	5.67 ± 0.40^a^
**Significance**			*	*	*	*	*	*
** LSD **_**0.05**_		M1	1.13	1.54	0.76	3.77	1.54	0.49
** LSD **_**0.05**_		M2	0.46	0.98	0.29	1.09	0.51	0.21
**Sids12**								
** Sids12**	**Control**	M1	98.3 ± 5.03^b^	5.33 ± 1.15^e^	13.7 ± 0.90^d^	18.0 ± 4.0^c^	3.0 ± 0.0^c^	4.1 ± 0.70^c^
		M2	100.5 ± 2.11^b^	7.0 ± 2.82^e^	13.14 ± 0.77^e^	19.0 ± 2.10^d^	2.7 ± 1.34^c^	3.98 ± 0.53^d^
** S**_**12**_** 129**	**0.0125 SA**	M1	98.5 ± 1.41^b^	24.0 ± 1.41^a^	18.1 ± 1.27^c^	26.0 ± 2.8^b^	6.5 ± 1.4^a^	5.7 ± 0.70^b^
		M2	97.1 ± 3.04^c^	25.4 ± 3.29^a^	21.02 ± 1.23^c^	26.4 ± 2.52^c^	5.0 ± 1.33^b^	5.35 ± 0.38^c^
** S**_**12**_** 144**	**0.025 SA**	M1	105.3 ± 6.2^a^	16.0 ± 2.0^d^	20.2 ± 2.22^b^	26.66 ± 2.3^b^	5.0 ± 2.0^b^	5.26 ± 0.80^b^
		M2	103.6 ± 0.99^a^	16.6 ± 2.69^d^	21.84 ± 0.66^b^	26..0 ± 2.66^c^	5.6 ± 1.03^a^	5.62 ± 0.69^b^
** S**_**12**_** 167**	**0.0125 HZ**	M1	98.2 ± 2.8^b^	22.0 ± 2.0^b^	24.5 ± 1.2^a^	28.0 ± 4.0^ab^	4.33 ± 1.15^b^	7.13 ± 1.22^a^
		M2	94.9 ± 1.01^d^	23.2 ± 2.06^b^	24.95 ± 0.89^a^	30.6 ± 3.29^a^	4.8 ± 1.57^b^	6.64 ± 0.35^a^
** S**_**12**_** 193**	**0.025 HZ**	M1	88.16 ± 2.1^c^	18.3 ± 1.15^c^	19.1 ± 1.31^bc^	30.0 ± 4.0^a^	6.33 ± 1.15^a^	6.0 ± 0.91^b^
		M2	90.8 ± 0.59^e^	19.4 ± 2.69^c^	20.03 ± 0.69^d^	28.2 ± 3.97^b^	5.1 ± 0.63^b^	6.73 ± 0.32^a^
**Significance**			*	*	*	*	*	*
** LSD **_**0.05**_		M1	3.83	1.48	1.35	3.29	1.20	0.84
** LSD **_**0.05**_		M2	0.83	1.19	0.41	1.32	0.48	0.20
**Giza168**								
** Giza168**	**Control**	M1	101.16 ± 1.72^b^	8.33 ± 1.15^b^	13.2 ± 0.80^b^	20.6 ± 2.30^b^	3.33 ± 1.15^b^	3.2 ± 0.70^b^
		M2	99.73 ± 0.90^b^	6.7 ± 1.34^b^	12.89 ± 0.53^b^	17.2 ± 2.07^b^	2.8 ± 1.26^b^	3.16 ± 1.29^b^
** G**_**168**_** 218**	**0.025 HZ**	M1	108.9 ± 1.6^a^	18.33 ± 3.05^a^	17.9 ± 1.10^a^	26.6 ± 2.30^a^	6.0 ± 2.0^a^	4.9 ± 0.80^a^
		M2	112.34 ± 0.73^a^	20.7 ± 1.34^a^	17.95 ± 0.87^a^	25.0 ± 2.11^a^	4.7 ± 0.97^a^	5.32 ± 0.56^a^
**Significance**			*	*	*	*	*	*
** LSD **_**0.05**_		M1	1.88	2.62	1.09	2.61	1.85	0.86
** LSD **_**0.05**_		M2	0.45	0.75	0.44	1.05	0.63	0.59

Each value is a mean of ten replicates

^*^Means significance at 0.05 levels of probability. Groups sharing the same alphabetical superscripts are not significantly different from each other. Groups with different superscripts indicate significant differences at the 0.05 probability level

On the contrary, Sids_12_ exhibited higher mean values for certain morphological and agronomical traits at M2 compared to the M1 generation. For example, the highest mean value of plant height (103.6 cm) was obtained in the S_12_144 genotype compared to the S_12_193 genotype, which exhibited the lowest value for plant height (90.8 cm). Moreover, the highest value for the average no. of grains /spikelet (5.6) was also obtained in S_12_144. The wheat genotype S_12_129 produced the highest no. of spikes /plant (25.4) on average. While the highest mean values for spike length (24.95 cm) and no. of spikelets /spike (30.6) were recorded in the S_12_167 mutated genotype, The highest mean 100-grain weight (6.73) was recorded for the S_12_193 mutated genotype compared with the control (Fig. 1 and Table 1).

Comparing both generations, Giza168 and one of its selected mutants produced the highest spikes /plant, spike length, no. of spikelets/spike, no. of grains/spikelet, and 100-grain weight in M2 compared with the M1 generation. Moreover, one mutant selected from the G168 218 genotype produced the highest mean values of plant height (112.34 cm), no. of spikes /plant (20.7), spike length (17.95), and 100-grain weight (5.32) compared with the control genotype (Fig. 1 and Table 1).

### The analysis of polymorphism using SCoT markers

**Fig. 2:**
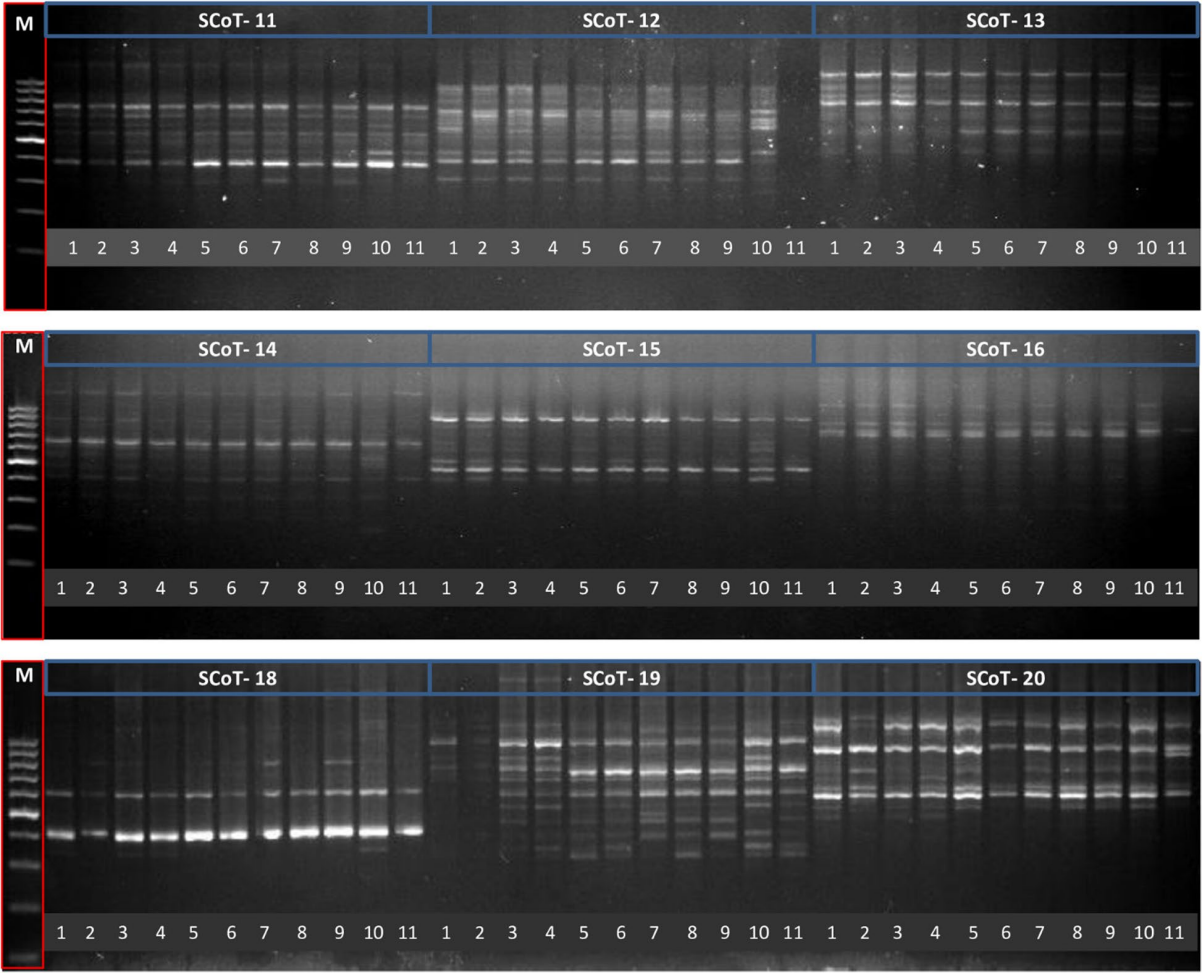
SCoT pattern using nine primers (e.g., SCoT-O11 to SCoT-O20) among eleven genotypes, (1) Sids1, (2) S 36, (3) S 83, (4) S 107, (5) Sids12, (6) S 129, (7) S 144, (8) S 167, (9) S 193, (10) Giza 168, (11) and G 218. M = molecular weight marker (100bp)

In this study, nine primers were used to analyze the genetic diversity of eleven wheat genotypes (Table 2). The amplification products generated by these primers exhibited polymorphic fingerprint patterns. 101 DNA fragments were acquired from nine primers, with a mean of 4.81 bands per primer (Figs. 2 and S1). Out of 101 amplified sections, only 41 were polymorphic, resulting in an estimated average of 1.95 polymorphic bands per primer. The nine primers used in this study exhibited a polymorphism level of 37.97%. The number of monomorphic bands seen was recorded as 60, with a mean value of 2.85 bands per primer. The primers SCoT-19 and SCoT-12 exhibited the highest level of polymorphism, with 8 and 5 polymorphic bands detected, respectively. The combinations SCoT-11, SCoT-13, SCoT-14, and SCoT-18 were shown to provide the minimum amount of amplified polymorphic fragments (Figure S1). The proportion of polymorphism increased from 16.7% in SCoT-11 to 100% in SCoT-19 and SCoT-12, as shown in Table 3.

**Table 2:** SCoT primer sequences that were used in this study. The sequence was retrieved from the NCBI database

No	Primer codes	Primer nucleotide sequence (5′ → 3′)	Annealing temperatures (^◦^C)
1	**SCoT-O11**	CAACAATGGCTACCACCA	53.7
2	**SCoT-O12**	CAACAATGGCTACCACCC	56.0
3	**SCoT-O13**	CAACAATGGCTACCACCG	56.0
4	**SCoT-O14**	CAACAATGGCTACCACGA	53.7
5	**SCoT-O15**	CAACAATGGCTACCACGC	55.6
6	**SCoT-O16**	CAACAATGGCTACCACGG	56.0
7	**SCoT-O18**	CAACAATGGCTACCAGCA	53.7
8	**SCoT-O19**	CAACAATGGCTACCAGCC	56.0
9	**SCoT-O20**	CAACAATGGCTACCAGGG	56.0

**Table 3:** Polymorphic and monomorphic bands of SCoT primers of the three wheat varieties and their mutant genotypes. Sids1 and their three mutant genotypes (S 36, S 83 and S 107), Sids12 and their four mutant genotypes (S 129, S 144, S 167 and S 193) and Giza 168 and its mutant genotype (G 218)

Primer Name	Genotypes	T	P	M	%P
**SCoT-11**	**S1**	7	2	5	28.5%
	**S12**				
	**G168**	6	1	5	16.7%
**SCoT-12**	**S1**	5	1	4	20%
	**S12**				
	**G168**	5	5	-	100%
**SCoT-13**	**S1**	4	2	2	50%
	**S12**	5	1	4	20%
	**G168**	4	3	1	75%
**SCoT-14**	**S1**	4	2	2	50%
	**S12**	3	-	3	0%
	**G168**	4	1	3	25%
**SCoT-15**	**S1**	4	2	2	50%
	**S12**				
	**G168**				
**SCoT-16**	**S1**	3	2	1	66.7%
	**S12**				
	**G168**				
**SCoT-18**	**S1**	2	-	2	0%
	**S12**	3	1	2	33.3%
	**G168**	2	-	2	0%
**SCoT-19**	**S1**	8	8	-	100%
	**S12**	6	-	6	0%
	**G168**	9	2	7	22.2%
**SCoT-20**	**S1**	6	4	2	66.7%
	**S12**	5	2	3	40%
	**G168**	6	2	4	33.3%
**Total**		101	41	60	
**Average**		4.81	1.95	2.85	37.97%

(*T*) The total bands, (*M*) monomorphic, (*P*) polymorphic, and (*%P*) percentage of polymorphic

### Clustering and genetic relatedness using SCoT markers

**Fig. 3:**
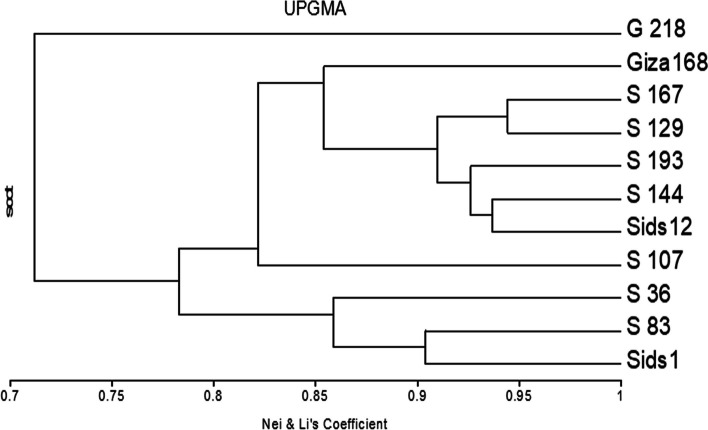
Dendrogram of three cultivars and eight selected mutant lines of wheat genotypes (e.g., Sids1, Sids12, Giza168, S 36, S 83, S 107, S 129, S 144, S 167, S 193 and G 218) generated by UPGMA cluster analysis of the dissimilarity values based on Nei’s coefficient [33]

The genetic similarity and clustering patterns of SCoT marker data from eleven genotypes have been investigated using the Unweighted Pair-Group Strategy employing Arithmetic Average (UPGMA) method and Dice coefficient, as shown in Fig. 3 and Table 4. The genetic similarity degree was assessed to be between 0.94 and 0.57, indicating a significant degree of proximity. The highest degree of genetic relatedness was observed within S12, S144, S12 167, and S12 129, with a value of 0.94. Conversely, the lowest level of genetic similarity was found throughout S1 36 and G218, with a value of 0.57. The UPGMA dendrogram exhibited a dichotomy whereby the initial cluster consisted of Sids1, S1 36, S1 83, and S1 107. The second cluster consisted of Sids12, S12 129, S12 144, S12 167, S12 193, and Giza168. The findings of this study demonstrate the efficacy of SCoT markers in identifying variation across various wheat genotypes and their corresponding mutant genotype lines (Fig. 3 and Table 4).

**Table 4:** Similarity coefficient values among the three studied cultivars (Sids1, Sids12 and Giza168) and the eight selected mutants derived from them (S36, S83, S107, S129, S144, S167, S193 and G218)

	**Sids1**	**S 36**	**S 83**	**S 107**	**Sids12**	**S 129**	**S 144**	**S 167**	**S 193**	**Giza168**	**G 218**
Sids1	1.00										
S 36	0.89	1.00									
S 83	0.90	0.83	1.00								
S 107	0.74	0.65	0.83	1.00							
Sids12	0.81	0.78	0.84	0.82	1.00						
S 129	0.76	0.76	0.84	0.82	0.91	1.00					
S 144	0.80	0.77	0.85	0.81	0.94	0.90	1.00				
S 167	0.75	0.76	0.82	0.82	0.93	0.94	0.92	1.00			
S 193	0.74	0.71	0.77	0.87	0.92	0.87	0.93	0.93	1.00		
Giza168	0.81	0.79	0.89	0.80	0.88	0.86	0.86	0.86	0.82	1.00	
G 218	0.61	0.57	0.70	0.72	0.74	0.77	0.71	0.81	0.75	0.72	1.00

Values are based on band polymorphisms generated by SCoT molecular markers

### Function Assessment of the SCoT-Associated Genes

To anticipate the biological functions of the nine SCoT-associated genes in question, the sequence of SCoT primers was searched against the genome sequence of *T. aestivum*. Subsequently, other databases like Ensembl Plants, Phytozome, National Center for Biotechnology Information (NCBI), InterPro, and Kyoto Encyclopedia of Genes and Genomes (KEGG) generated further functional annotations for these genes. In the given context, it can be shown that these nine genes are linked to the ABSCISIC ACID INSENSITIVE3 (ABI3)/VIVIPAROUS1(VP1)(RAV) transcription factors. These transcription factors are characterized by the presence of a B3 domain and an APETALA2 (AP2) domain, and they are categorized under the APETALA2/ethylene-responsive element binding factor (AP2/ERF) or B3 superfamily. These are potentially linked to vital biological functions, such as responding to environmental stressors and controlling plant growth and development. Similar findings of these transcription factors being associated with essential plant traits have also been reported in *Arabidopsis thaliana*, *Zea mays*, *Oryza sativa*, *Glycine max*, *Solanum lycopersicum*, *Medicago truncatula*, and *Capsicum annuum* [34–39].

### Putative Tissue Expression Patterns of the SCoT-Associated Genes

**Fig. 4:**
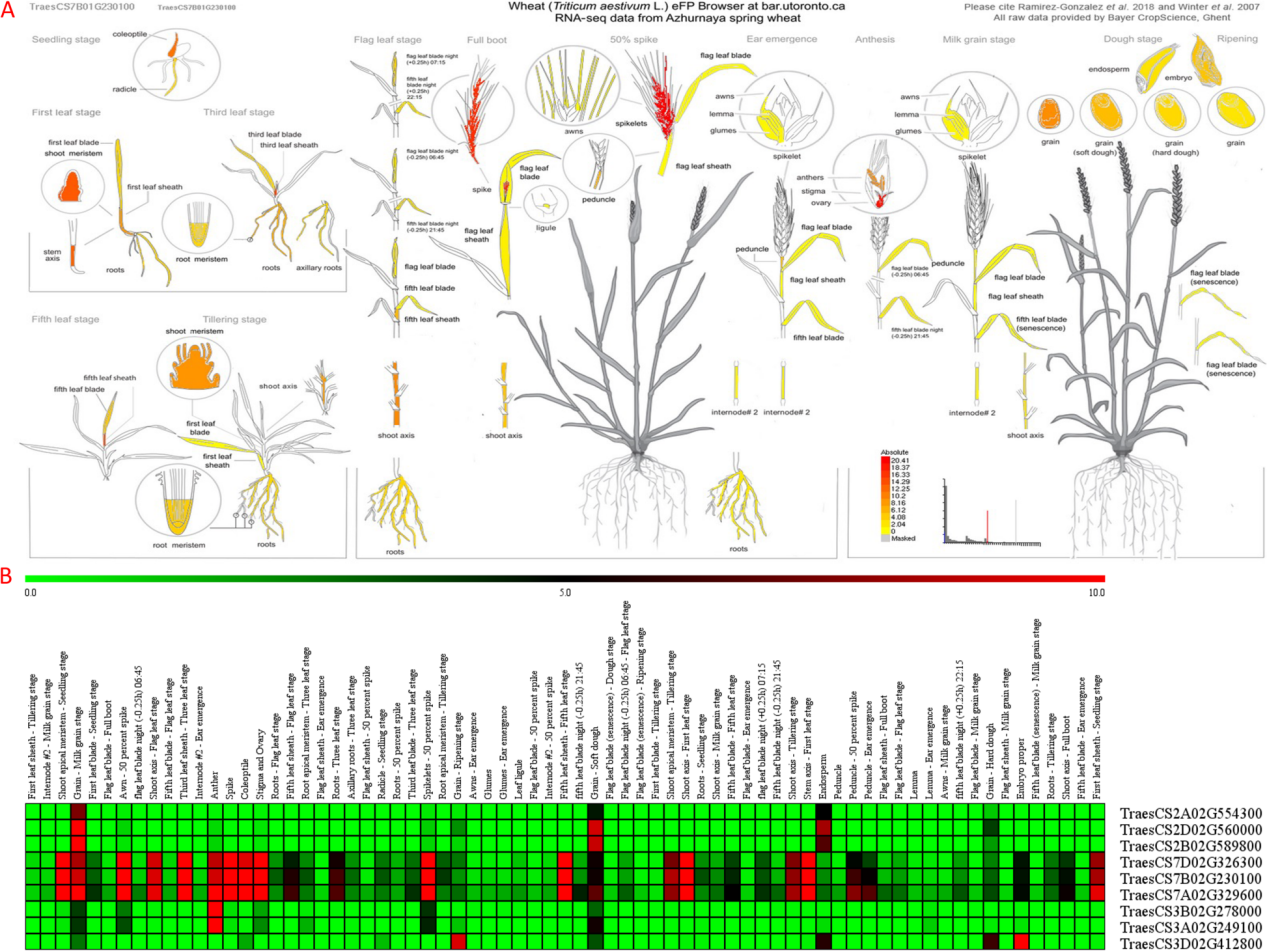
The putative “wheat electronic fluorescent pictograph” tissue expression of TraesCS2A02G554300, TraesCS2D02G560000, TraesCS2B02G589800, TraesCS7D02G326300, TraesCS7B02G230100, TraesCS7A02G329600, TraesCS3B02G278000, TraesCS3A02G249100 and TraesCS3D02G412800 genes at different tissues and developmental stages. The more intense the red color of the expression bar, the more gene expression is detected

To comprehend the potential roles of the nine SCoT-associated wheat genes in different tissues, their expression patterns were analyzed using the *T.* *aestivum* transcript expression database (Fig. 4). The findings revealed varied expression patterns of the genes TraesCS7D02G326300, TraesCS7B02G230100, and TraesCS7A02G329600 across numerous wheat tissues, with particularly prominent expression observed in spikelets (50 percent spike), stigma, and ovary. Additionally, notable expression was detected in the awn (50 percent spike), third leaf sheath (at the three-leaf stage), fifth leaf sheath (at the fifth leaf stage), spike, shoot apical meristem (at the seedling stage), shoot axis (at the first leaf stage), coleoptile, and first leaf sheath (at the seedling stage), as illustrated in Fig. 4 and Table S2. Additionally, the highest expression levels for the genes, TraesCS2A02G554300, TraesCS2D02G560000, and TraesCS2B02G589800, were recorded at the grain (milk grain), grain (soft dough), endosperm, and grain (hard dough). Moreover, the two genes, TraesCS3B02G278000 and TraesCS3A02G249100, were highly expressed in anther, grain (soft dough), grain (milk grain), Spikelets (50 percent spike), awn (50 percent spike), stigma, ovary, and spike. In comparison, the TraesCS3D02G412800 gene exhibited high expression in the embryo proper, grains of the ripening, hard dough, milk grain, and soft dough stages, and the coleoptile and radicle of the seedling stage (Fig. 4 and Table S2).

## Discussion

In nature, assessing morphological and agronomical traits is critical for distinguishing and identifying mutant genotypes with desirable traits developed via mutagenesis. Herby, the morpho-agronomic characters for three varieties of bread wheat and their mutants at M1 and M2 generations, as affected by specific concentrations of SA and HZ, were assessed. M1 generations showed yield improvements compared to conventional wheat varieties, but most importantly, they provided a platform for rapid screening and identification of lines with desirable agronomic traits. We further evaluated these lines for stability and heritability in M2. It was found that M2 had higher estimates of morphological and agronomical traits than M1 and the control; this suggests that the observed heterogeneity has a strong genetic basis (Fig. 1 and Table 1). These improved M2 traits concurred with other documented experiments in which the mean values of 100-grain weight for *H. vulgare* were higher in M2 generations than in M1 [40].

According to other findings, mutagen treatment showed a more positive effect on the elongation of spike length in M2 generations than in M1 and original *T. aestivum* varieties [41]. In addition, a notable disparity was seen in many features, including the number of spikelets per spike, spike length, and plant height, after mutagen treatment in the M2 generation of *T. aestivum*, as compared to the M1 generation [42–44]. This discrepancy can be attributed to the possibility that specific mutations may alter the protein structure. For example, a mutation might lead to a protein with a slightly different conformation, resulting in reduced effectiveness compared to the wild-type protein. Mutations within the protein-coding region can alter the amino acid sequence, while mutations in other regions may impact the transcriptional and translational regulation of gene expression [45]. Moreover, some studies recorded the top averages of some morphological and agronomical attributes at M2 generation compared with M1 generation under the effect of various mutation components such as spike length and hundred-grain weight gains [26, 46], a higher no. of grains/spikelet and spikelet number/spike in wheat [26], and a significant increase in no. of grains/spikelet in wheat compared with control plants [47–49].

Initially, the current study provides support for the utilization of the SCoT marker due to its simplicity and higher reproducibility compared to RAPD and ISSR techniques [50]. Secondly, the SCoT marker system reveals polymorphisms in protein-encoding regions due to the specificity of SCoT primers to amplify DNA sequences from the conserved area around the ATG translation start codon which in turn may improve our knowledge of the functional implications of genetic diversity [51, 52]. The amplified DNA regions from the SCoT marker approach may be associated with specific traits. SCoT markers can detect the presence or absence of dominant markers caused by co-dominant markers and sequence variations [50].

The genetic diversity in the eleven genotypes was assessed using nine SCoT primers, revealing a polymorphism level of approximately 20%. The bands per primer had a notably higher mean of 100%. These results align with prior research on 14 wheat cultivars from North Africa, where observed polymorphism levels ranged from 8 to 57%, with a typical rate of 34.5% [53]. Similarly, 30 (SCoT) primers were used to investigate the genetic diversity among eight Asian wheat cultivars. The analysis revealed an average polymorphism rate of 38.4% [54].

Moreover, the SCoT markers have been extensively employed in research to analyze the polymorphism percentage levels at various plants and crops such as sweet potato (56.5%) [55], peanut (38.22%) [51], mango (73.82%) [56], *Cicer* species (100%) [57], *Triticum urartu* (100%) [58], rice (83.26%) [30], and durum wheat (72.22%) [59]. On the other hand, various studies found that a change in a single nucleotide within or at the end of the SCoT primer might affect the banding pattern. The present study further reported that these primers, SCoT-O11, SCoT-O12, and SCoT-O13, differ only in the last nucleotide. In contrast, the primers SCoT-O14, SCoT-O15, and SCoT-O16 differ only in the last two nucleotides. The sequences of SCoT-O18, SCoT-O19, and SCoT-O20 only differ in the last three nucleotides from the other primers (Table 2). Because of these nucleotide differences, the SCoT primers make different DNA marker profiles [52, 55, 58].

The inorganic compounds HZ (N_2_H_4_ × H_2_O) and SA (NaN_3_) have significant importance, particularly in the field of agrochemicals, where they mainly serve as herbicides [60]. They are also classified as alkylators, such as sulfur mustards, alkyl methane sulphonates, and alkyl nitrosoureas. Alkylators are chemical mutagens that can induce mutations [9, 13]. Chemical mutagens have been extensively employed to modify the genetic composition of plants in various manners, such as altering nucleotides, disrupting DNA replication, introducing indels during DNA replication, and cross-linking two DNA nucleotides, resulting in single nucleotide polymorphisms (SNPs) [61]. Furthermore, it has been shown that chemical mutagens exhibit a higher level of mutation initiation and are readily employed in in vitro experiments as opposed to physical and radiation methods [62–65]. Certain checkpoints, such as nucleotide excision repair (NER) and base excision repair (BER), are necessary for the cells to repair mutations during cell divisions [65]. The impairment of these repair mechanisms has the potential to induce irreversible mutations that will have the capacity to modify gene expression and protein encoding, thereby generating genetic diversity that may contribute to enhanced crop productivity.

Inducing and selecting these favorable mutations has been done in mung bean (*Phaseolus aureus* Roxb.) [61, 66]. Another alkylating agent, EMS, is extensively utilized as a chemical mutagen in plants. EMS has been shown to elicit GC → AT transitions in the genomic DNA, producing mutant proteins that exhibit distinct functionalities compared to the wild-type protein [61]. Previous studies have provided evidence supporting the practicality and efficacy of alkylating chemicals, including EMS, SA, and HZ, to evolve unique gene pools in plants [11, 61, 66–69]. Hence, it may be inferred that alkylating chemicals are often used as agents for inducing point mutations, a genetic alteration characterized by modifying, adding, or deleting a single nucleotide base within an organism's DNA or RNA sequence [70]. The impacts on protein function, composition, and synthesis might vary depending on the kind of point mutation. These alterations include a spectrum of outcomes, ranging from beneficial consequences, as shown in synonymous mutations, to detrimental ones, as seen in nonsynonymous mutations [71, 72]. In the present context, these mutations can potentially induce diverse consequences for protein expression levels. For instance, they eliminate or insert a stop codon, leading to an abnormal extension or truncation of the translated protein.

Additionally, these mutations can induce alterations in the amino acid’s chemical and physical characteristics. Consequently, the affected polypeptide may experience a loss of function, acquire a novel function, or become activated [73, 74]. For example, in investigating the durability of mutations in aromatic amino acid (AAA) in *A. thaliana* seeds, the seeds were subjected to mutagenesis through EMS [75, 76]. The researchers successfully identified 351 mutants known as suppressors of *tyra2* (sota), which lack one of the two *TyrA* genes that biosynthesize tyrosine. These genes provided a shared substrate for the shikimate pathway [76]. The mutant under EMS effect investigation exhibited elevated levels of aromatic amino acids (AAAs) compared to other amino acids in the F1 and F2 generations of the plant population, which possessed dominant or semi-dominant traits. This increase in AAAs was concomitated with enhanced net CO_2_ fixation and increased shikimate pathway activity [75–78]. The findings presented in this study provide genetic support for the notion that harnessing chemical mutagens to induce point mutations might effectively and significantly augment plant performance.

Herein, introducing either SA or HZ leads to random point mutations. Subsequently, these mutations stimulate DNA repair mechanisms and pathways. Upon administering a specific dosage, this activation initiates an adaptive response that fortifies resistance against agents causing DNA damage, consequently reducing mutagenesis frequency [79, 80]. Also, induced mutagenesis can induce genes associated with photosynthesis, growth, development, and ABA signaling pathways [81]. Accordingly, our selected SCoT markers have exhibited observed correlations with key transcription factors, specifically ABSCISIC ACID INSENSITIVE3 (ABI3) / VIVIPAROUS1 (VP1) belonging to the RAV family. Given that RAV transcription factors play a pivotal role upstream of numerous protein-encoding genes responsible for regulating plant growth, development, and responses to both biotic and abiotic stressors [82], these correlations hold significant potential implications in the context of marker-assisted breeding programs or future biological pathway analysis. Also, it provides a rationale for the traits associated with yield.

## Conclusions

In conclusion, this study investigates how well mutagenesis, specifically treatments with SA and HZ, can improve traits in different types of wheat. We applied selective breeding techniques using SCoT markers to select traits within the mutant wheat gene pools. The study revealed an increase in agronomical characteristics in M2 genotypes compared to M1. Bioinformatics analysis also found a link between SCoT markers and transcription factors ABI3 and VP1, which are essential for plant development, growth, and stress management. This work sheds light on how chemical mutagens may be used to improve various traits in wheat, as well as how mutagenesis-induced changes occur in wheat, by examining the relationship between SCoT markers and certain agronomic traits. It may also aid in the proper selection of genetic resources in the development of new cultivars.

## Materials and methods

### Field experiment

The seeds of three Egyptian cultivars of bread wheat (*Triticum aestivum*)*,* which are Sids1, Sids12, and Giza168, were obtained from the Field Crops Research Institute (FCRI), Agricultural Research Centre (ARC), Giza, Cairo, Egypt. The selection of these varieties is rooted in their widespread cultivation in Egypt, their high productivity, and their resilience to adverse conditions [26, 83, 84]. Table 5 shows the lineage and origin of these genotypes. Dry seeds (~ 100 grains/treatment) were immersed in distilled water for 10 h before being soaked in three different concentrations of SA and HZ (i.e., 0.0, 0.0125, and 0.025) for 12 h in season 2019/2020. Then, the treated seeds were rinsed in distilled water for two hours. Finally, treated and untreated seeds were planted in the soil. The field trial for the M1 generation was acquired at a private farm in Alexandria Governorate, Egypt, in the 2019–2020 season. The experimental plot was 2.5 m long and 20 cm apart and was assigned under Randomized Complete Block Design (RCBD). Plants were maintained in the field under regular day/night, irrigation, and fertilization conditions. At the end of the first season, eleven mutants were selected based on the higher morphological and agronomical traits (e.g., plant height, no. of spikes/plant, spike length, no. of spikelet/spike, no. of grains/spikelet, 100-grain weight) compared with the control (Table 6). In addition, we excluded a few mutants based on the lower morphological and agronomical traits that were recorded compared with the control (e.g., plant height, no. of spikes/plant, spike length, no. of spikelet/spike, no. of grains/spikelet, 100 grain weight) (Table S1). To achieve M2 generation, the three original varieties and eight selected mutant genotypes from M1 were sown on the same farm during 2020–2021.

**Table 5:** The Pedigree and origin of the three selected bread wheat (*Triticum aestivum*) varieties

Name	Pedigree	Origin
Sids1	MRL/BUC/SER1	Egypt
Sids12	BUC//7C/ALD/5/MAYA74/0N//1160 Egypt/47/3/BB/GLL/4/CHAT"S"/6/MAYA/VUL—//CMH74A.63014*SX.SD7096-4SD-1SD-1SD-0SD	Egypt
Giza168	MRL / BUC // SERI – CM 930 46- 8 M-OY-OM-2Y-OB-OG	Egypt

**Table 6:** The Eleven bread wheat (*Triticum aestivum*) genotypes used in this study: Sids1 and their three mutant genotypes (S 36, S 83 and S 107), Sids12 and their four mutant genotypes (S 129, S 144, S 167 and S 193) and Giza 168 and its mutant genotype (G 218)

Variety	Sr. No	Genotype Code	Treatment
Sids1	1	Sids1	Control (0.0%)
	2	S_1_ 36	0.025% SA
	3	S_1_ 83	0.0125% HZ
	4	S_1_ 107	0.025% HZ
Sids12	5	Sids12	Control (0.0%)
	6	S_12_ 129	0.0125% SA
	7	S_12_ 144	0.025% SA
	8	S_12_ 167	0.0125% HZ
	9	S_12_ 193	0.025% HZ
Giza168	10	Giza168	Control (0.0%)
	11	G_168_ 218	0.025% HZ

*Sr. No.* Serial Number, *SA* Sodium Azide, *HZ* Hydrazine Hydrate

### Assessing agronomic traits

We harvested the wheat plants during the 2019–2020 growth season after they had grown for seven months. We then randomly selected ten plants from each replication of each treatment for further investigation. We meticulously recorded various agronomic traits during this period, including plant height, the number of spikes per plant, spike length, the number of spikelets per spike, the number of grains per spikelet, and the weight of 100 grains. Afterward, during the 2020–2021 growth season, the seeds from the M1 generation were sown, and after seven months of growth, the M2 plants were harvested. We then assessed the same agronomic traits.

### DNA extraction and quantification

Total genomic DNA was isolated from young wheat leaves using a Qiagen, Inc. DNEASY PLANT MINI KIT. The ND-1000 spectrophotometer (Nanodrop Technologies, USA) was used to quantify the extracted DNA. The *A*_260/280_ was used to check its quality. Then, its quantity and concentration were estimated [31, 32]. The genomic DNA samples were stored at –20 °C.

### PCR analysis

Table 2 shows the nine SCoT primers used in the present study. DNA amplification was carried out in a 25 μL volume comprising a 12.5 μL Master Mix (Sigma), 2.5 μL primer (10 pcmol), 2.5 μL of the DNA template (10 ng), and 7.5 μL dH_2_O for PCR amplification. The temperature profile for PCR analysis in an Eppendorf™ Mastercycler™ Nexus Thermal Cycler (Eppendorf North America, USA) comprised 34 cycles following a 4-min denaturation cycle at 95 °C. Each cycle includes a denaturation stage at 94 °C for 1 min of SCoT-PCR, an annealing stage at 53.7–56.0 °C (according to the selected primer) for 50 s of SCoT-PCR, and a prolonging stage at 72 °C for 1:30 min of SCoT-PCR. The final cycle extended the expansion phase to 6 min at 72 °C. The PCR products were electrophoresed at 100 V in a 1.8% agarose gel containing ethidium bromide (0.6 μg/mL) in a 1X TAE buffer. To assess the size of individual DNA bands, the DM3100-ExcelBand™ 1 KB (0.25–10 kb) DNA Ladder was utilized as a reference. Finally, the BIO-RAD gel documentation system (Gel Doc XR + System) was used to photograph the gel under ultraviolet light, and the patterns of amplified DNA were analyzed using the BIO-RAD software.

### SCoT primers

Twenty SCoT primers designed for wheat DNA genetic diversity analyses were utilized. These SCoT primer sequences were developed [52, 55, 58, 85], and nine out of twenty SCoT primers were used in the genetic diversity analysis of wheat genotypes (SCoT-O11, SCoT-O12, SCoT-O13, SCoT-O14, SCoT-O15, SCoT-O16, SCoT-O18, SCoT-O19, and SCoT-O20) based on producing precise and distinct banding patterns (Table 2).

### Data analysis

The analysis of variance (one-way ANOVA) and Duncan's multiple range tests at a 5% probability level were employed to evaluate the significance of differences between treatments. The statistical analysis of the obtained data was performed using the Costat software [86]. In the characterization of the SCoT fragments that were PCR-amplified and detected on gels, PCR-amplified fragments were denoted as '1' for signifying group proximity and '0' for the absence of such proximity. Subsequently, Jaccard's similarity coefficients were calculated using MVSP 3.2 software to construct a dendrogram based on these assignments. The Unweighted Pair-Group Strategy employing Arithmetic Average (UPGMA) was used as the clustering algorithm for dendrogram construction [87].

### Function predictions of wheat genes-associated SCoT markers

The SCoT marker sequence was utilized to query the *T. aestivum* genomes we got from the NCBI website database (https://www.ncbi.nlm.nih.gov/genome/11; retrieved on January 2, 2022). Then, the alignment sequence was compared using the available data from multiple NCBI GenBank, Phytozome, and Ensembl Plants databases to determine wheat SCoT primer candidate genes. Phytozome v13 and Ensembl Plants were used to derive annotations for these genes' probable roles [31, 32]. NCBI blast sequences were produced against the genomes of different wheat species: *T. aestivum*, *T. turgidum*, *T. dicoccoides*, and *T. urartu*.

### Potential tissue expression pattern of the target genes

In various tissues, we investigated the potential differential expression of the target genes (TraesCS2A02G554300, TraesCS2D02G560000, TraesCS2B02G589800, TraesCS7D02G326300, TraesCS7B02G230100, TraesCS7A02G329600, TraesCS3B02G249100, and TraesCS3D02G412800). We have linked these genes to the selected SCoT markers. We analyzed their expressions from the *T. aestivum* transcript expression database, which contains data on seventy-one different tissues and organs. Seedling stage: coleoptile, radicle, and roots. The first leaf stage includes the leaf blade, shoot apical meristem, leaf sheath, stem axis, roots, and leaf ligule. Three-leaf stage: third leaf sheath, root apical meristem, roots, axillary roots, and third leaf blade. Five-leaf stage: fifth leaf sheath and fifth leaf blade. Tillering Stage: first leaf sheath, root apical meristem, first leaf blade, shoot apical meristem, shoot axis, and roots. Flag leaf stage: shoot axis, flag leaf blade, fifth leaf blade, fifth leaf sheath, roots, flag leaf blade night (+ 0.25 h) at 07:15, fifth leaf blade night (+ 0.25 h) at 22:45, flag leaf blade night (-0.25 h) at 06:45, and fifth leaf blade night (-0.25 h) at 21:45. Full boot stage: flag leaf blade, flag leaf sheath, and shoot axis. Fifty percent spike stage: awn, flag leaf sheath, roots, spike, spikelets, flag leaf blade, internode #2, and peduncle. Ear emergence stage: internode #2, flag leaf sheath, awns, glumes, flag leaf blade, peduncle, lemma, and fifth leaf blade. Anthesis stage: anthers, stigma, and ovary; flag leaf blade (-0.25 h) at 06:45; and fifth leaf blade night (-0.25 h) at 21:45. Milk grain stage: awns, lemma, glumes, peduncle, flag leaf blade, flag leaf sheath, fifth leaf blade (senescence), internode #2, grain, and shoot axis. Dough stages include grain (soft dough), grain (hard dough), flag leaf blade (senescence), and endosperm. Ripening stage: embryo proper, grain, and flag leaf blade (senescence). We generated expression profiles for the wheat plant using Electronic Fluorescent Pictograph Browsers (Wheat eFP browsers; https://bar.utoronto.ca/efp_wheat/cgi-bin/efpWeb.cgi; accessed on January 5, 2022) [31, 32].

### Supplementary Information

Supplementary Material 1.

Supplementary Material 2.

Supplementary Material 3.

## Data Availability

All data generated in this study are available from the corresponding author on reasonable request.

## Abbreviations

ABI3: ABSCISIC ACID INSENSITIVE3
AFLP: Amplified Fragment Length Polymorphism
ANOVA: Analysis of Variance
ARC: Agricultural Research Centre
BER: Base Excision Repair
DNA: Deoxyribonucleic Acid
EMS: Ethyl Methane Sulphonate
FCRI: Field Crops Research Institute
HZ: Hydrazine Hydrate
ISSR: Inter Simple Sequence Repeat
Kb: Kilobase
KEGG: Kyoto Encyclopedia of Genes and Genomes
M1: First Generation
M2: Second Generation
NCBI: National Center for Biotechnology Information
NER: Nucleotide Excision Repair
Nm: Nanometers
no.: Number
PCR: Polymerase Chain Reaction
RAPD: Random Amplified Polymorphic DNA
RCBD: Randomized Complete Block Design
RFLP: Restriction Fragment Length Polymorphism
RNA: Ribonucleic acid
SA: Sodium Azide
SCoT: Start Codon Targeted
SNP: Single Nucleotide Polymorphism
SSR: Simple Sequence Repeat
UPGMA: Unweighted Pair-Group Strategy employing Arithmetic Average
VP1: VIVIPAROUS1
µg: Micrograms
