# Supplementary material for: Induced genetic diversity through mutagenesis in wheat gene pool and significant use of SCoT markers to underpin key agronomic traits
Source: BMC Plant Biol. 2024 Jul 15;24:673. doi: 10.1186/s12870-024-05345-5 (PMC11247860; doi:10.1186/s12870-024-05345-5)
Supplement: Supplementary file 3 — Supplementary Material 3. [file 12870_2024_5345_MOESM3_ESM.docx]

SCoT ( O-11, O-12, O-13 )


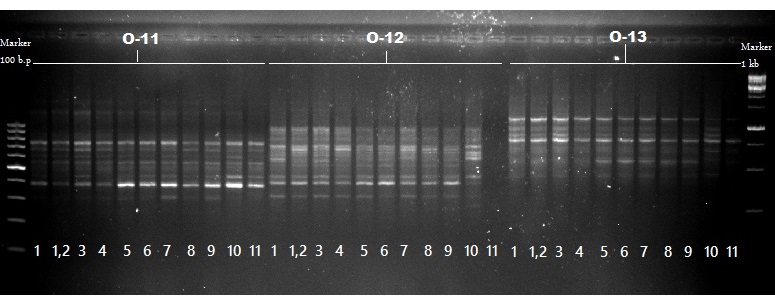


SCoT ( O-14, O-15, O-16 )


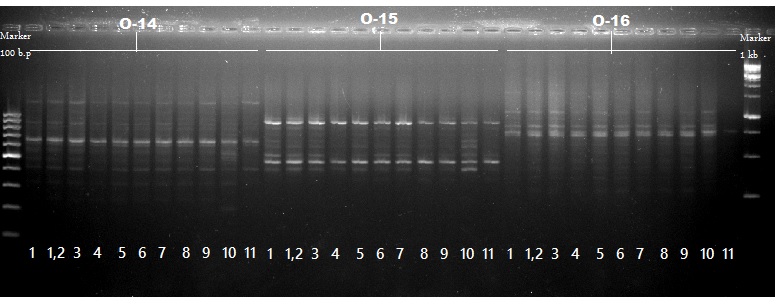


SCoT ( O-18, O-19, O-20 )


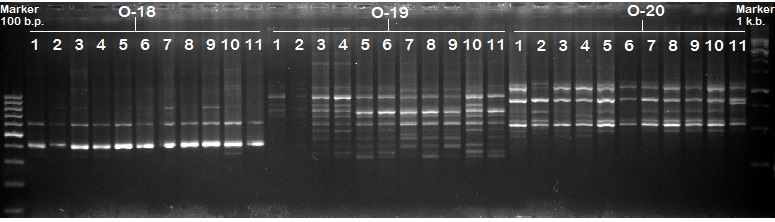


**Figure S1**. PCR profile of nine SCoT primers (SCoT-O11 to SCoT-O20) among eleven wheat genotypes. M = DNA size marker (100bps), Lanes 1-11 DNA samples of Sids1, S 36, S 83, S 107, Sids12, S 129, S 144, S 167, S 193, Giza168, and G 218, respectively.
